# The professional role of massage therapists in patient care in Canadian urban hospitals – a mixed methods study

**DOI:** 10.1186/s12906-015-0536-4

**Published:** 2015-02-07

**Authors:** Ania Kania-Richmond, Barb Findlay Reece, Esther Suter, Marja J Verhoef

**Affiliations:** Department of Community Health Sciences, Faculty of Medicine, University of Calgary, Calgary, AB Canada; Workforce Research & Evaluation, Alberta Health Services, Calgary, AB Canada; Association of Registered Nurses of British Columbia, Vancouver, BC Canada

**Keywords:** Massage therapy, Role, Hospital

## Abstract

**Background:**

Massage therapy (MT) is becoming established as a recognized health care profession in Canada. It has been integrated as a core service in settings such as health spas, private integrative health centers, and there is indication that MT is starting to be integrated into hospitals. Research in the area of hospital-based MT has primarily focused on the efficacy, effectiveness, and increasingly, the safety of MT. However, little is known about the professional role of massage therapists in the hospital setting. The purpose of this study was to conduct an in-depth exploration and description of massage therapists’ professional role in patient care in the context of Canadian urban hospitals.

**Methods:**

A sequential mixed methods study design was used. For the quantitative phase, a survey was sent to urban hospitals where MT services were organized by hospitals and provided by licensed massage therapists to patients to a) provide a contextual description of the hospitals and b) identify a sampling frame for the qualitative phase. The subsequent qualitative phase entailed semi structured interviews with a purposively diverse sample of participants massage therapists from the surveyed sites to explore their role perceptions. The quantitative and qualitative approaches were integrated during data collection and analysis.

**Results:**

Of the hospitals that responded, sixteen urban hospitals across Canada (5%) provided MT to patients by licensed therapists. The majority of hospitals were located in Ontario and ranged from specialized small community hospitals to large multi-site hospitals. Based on interviews with 25 participants, six components of the massage therapists’ professional role emerged: health care provider, team member, program support, educator, promoter of the profession, and researcher.

**Conclusions:**

While hospital-based MT in Canada is not a new phenomenon, MT is not yet an established health care profession in such settings. However, there is significant potential for the inclusion of the massage therapists’ role in Canadian hospitals that should be evidence based for effective implementation.

**Electronic supplementary material:**

The online version of this article (doi:10.1186/s12906-015-0536-4) contains supplementary material, which is available to authorized users.

## Background

Massage therapy is broadly defined as the manual manipulation of soft body tissues to enhance health and well-being [1]. Massage therapy services are used by patients and health consumers to address a wide spectrum of health related issues, ranging from improving general wellness to supporting the treatment of specific conditions. They either access these services directly or are referred to them by other health care professionals (HCPs).

Within the Canadian context, massage therapy is increasingly recognized as a health care profession supporting the health of Canadians, and is a regulated health care profession in five provinces (British Columbia, Manitoba, Ontario, New Brunswick, and Newfoundland). In these provinces, the scope of massage therapy practice is: “the assessment of the soft tissues and joints of the body and the treatment and prevention of physical dysfunction and pain of soft tissue and joints by manipulation to develop, maintain, rehabilitate or augment physical function or to relieve pain” [2-4]. Massage therapy has been integrated as a core service in settings such as health spas and private integrative health centers. Although its presence and acceptance as a therapeutic intervention within the current Canadian health care system has been variable, there are indicators that massage therapy is starting to be integrated into hospitals.

Research in the area of hospital-based massage therapy has primarily focused on the efficacy, effectiveness, and increasingly, the safety of massage therapy [5,6]. This research is crucial to support the integration of massage therapy into conventional biomedical health care settings [7-9]. The effective integration of massage therapy into these health care settings depends on a number of factors, such as acceptance and evidence of effectiveness. One key factor that has received little attention is how the massage therapist’s professional role is understood – by the massage therapist and others. A professional role is defined as a cluster of recognizable behaviours displayed by the role occupant in carrying out various tasks, functions, and responsibilities [10,11]. A role-focused inquiry provides important information regarding the way massage therapists function within a health care setting, the tasks and activities they are expected to carry out, the responsibilities they assume, and how their work is defined in relation to other health care professional (HCPs). Such knowledge is essential for the effective integration of massage therapy in patient care in hospital settings. To date, no systematic data have been collected on massage therapy provision in hospitals. Only one study was identified that explored issues related to the professional status of massage therapists in the context of interprofessional collaboration in a hospital setting [12]. The findings of this study indicate that the massage therapists’ position within the hierarchical structure of the hospital setting is highly constrained and generally excluded from the interprofessional milieu. This may result from other health professionals’ limited knowledge about massage therapy as a health care profession (within the Canadian context) and their negative associations with the term “massage”. The findings of this study are limited in terms of being generated from one hospital setting and not identifying the specific functions, responsibilities and position of massage therapists within interprofessional settings; however, it does demonstrate the importance of context in generating a complete understanding of professional roles. As an evolving profession, in which massage therapists are increasingly required to negotiate their professional boundaries in multi-professional biomedical settings, a clear delineation of their professional role is particularly relevant and needed.

The purpose of this study was to conduct an in-depth exploration and description of massage therapists’ professional role in patient care in the context of Canadian urban hospitals.

The objectives were to:

1. Describe the hospitals within which massage therapists’ roles are enacted.
2. Explore and describe the role of licensed massage therapists in Canadian urban hospital settings, as perceived by massage therapists who provide patient care in hospitals.
3. Explore if and how massage therapists’ perceptions of their role vary across the hospitals.

## Methods

### Study design

A sequential mixed methods (quantitative and qualitative phases) study design [13,14] was used to address the study objectives. The quantitative phase consisted of a descriptive survey of urban hospitals (the study sites) that delivered massage therapy services to patients by licensed massage therapists to a) provide a contextual description of these hospitals and b) identify a sampling frame for the qualitative phase. The subsequent qualitative phase, which was the main focus of the study, consisted of semi-structured in-depth interviews with massage therapists who worked at the hospital and provided patient care to explore their role perceptions. The quantitative and qualitative approaches were integrated during data collection and analysis.

This study design was most appropriate to address the study objectives for several reasons. First, the lack of research on this topic indicated the need for an exploratory phase to generate an understanding of the massage therapists’ professional role. Second, the survey provided context by describing the settings where massage therapists delivered care. Lastly, the breadth of the findings was expanded by comparing thematic findings across contexts.

A systematic search was conducted to identify hospitals across the 33 Canadian urban centers (see Additional file 1) where massage therapy is provided by licensed massage therapists. Once a list of all hospitals in the 33 urban centers was compiled, two approaches were used to identify hospitals providing massage therapy services. First, by reviewing hospital websites and directly contacting the hospital’s general information line by email or phone call to inquiry about massage therapy provision. Second, by circulating a research notice to massage therapists across Canada, requesting for those who worked in a hospital setting to contact research personnel. The research notice was circulated by enlisting the assistance of massage therapy professional associations and regulatory bodies across Canada (see Additional file 2). Each of these organizations provided explicit agreement to circulating the notice to their respective memberships.

The study was conducted at the University of Calgary. Ethics approval was obtained from the Conjoint Research Ethics Board at the University of Calgary (Ethics ID: E-24230). Consent was implied by responding to a recruitment email. In addition, verbal consent was obtained from each participant prior to the initiation of a survey or an interview.

### Data collection

#### Survey

Data specific to the description of hospitals (objective 1) were collected using Section 1 of the Hospital-Based Massage Therapy Questionnaire (HBMTQ), which was developed for the purposes of this study (see Additional file 3). Face validity was established through cognitive interviews with experts in the area of massage therapy and complementary and alternative medicine (n = 7), and hospital administrators (n = 2). The survey was administered as a structured telephone interview by the researcher (AKR) and completed by a manager or director of the hospital ward where massage therapy was incorporated when available or by one of the massage therapists. The option to complete the questionnaire independently and return it to the researcher by fax or email was also available so as not to limit the inclusion of any potential study site. The survey phase was completed between January 2012 and March 2012.

#### Interviews

Semi-structured in-depth interviews were conducted to explore and describe perceptions of the massage therapists’ role (see Additional file 4). Interviews were conducted by telephone with licensed massage therapists who provided patient care in Canadian urban hospitals. A maximum variation sampling strategy [14] was used to generate the sample of massage therapist participants from across the study sites. Interviews were completed between February 2012 and April 2013. Interviews lasted between 20 to 60 minutes. Key topics addressed were related to massage therapists’ activities, functions, and responsibilities (who determines what massage therapists do; role overlap; and, expectations of massage therapists).

### Data analysis

#### Survey

All completed questionnaires were de-identified and cleaned, and verified data were entered into Microsoft Excel. Descriptive statistics, such as counts and percentages, were obtained for all variables. Further inferential analyses were not conducted as the main purpose of the quantitative phase was descriptive; namely, to contextualize the interview data and to identify the sampling frame for the qualitative phase. Furthermore, the small sample size precluded comparative analyses across study sites that could be statistically evaluated.

#### Interviews

All interviews were digitally recorded and transcribed verbatim. NVIVO 9.0 was used to manage the qualitative data and facilitate the analysis. The analysis procedure was based on qualitative content analysis [15-17], which is a systematic method to analyze textual data into content categories (codes and themes). The codes and themes were determined inductively, emerging out of the data during the analysis [16,17].

#### Mixed analysis – contextualizing the qualitative findings

A descriptive matrix [18-20] was used to display and examine, in a descriptive manner, if and how the emergent themes that identified the MT role varied across study sites. Such an analytic approach is useful in showing similarities, differences, and patterns that enables a rich understanding of the phenomena under study. Themes were transformed by “quantitization of qualitative data” [13,14] and included in the matrix. In this process, qualitative data are converted, or transformed, into quantitative (or dichotomous) categories. Here, each theme was coded as present or not present in the matrix.

The analysis was descriptive only as the low number of study sites precluded comparisons that could be statistically evaluated. Although not conclusive, such comparisons do provide useful insights as to how the massage therapists’ role may vary in relation to the context within which it is enacted.

## Results

The study consisted of two inter-connected phases. The first (quantitative) phase consisted of a survey; the sample for this phase included 15 hospitals where massage therapy is provided by licensed massage therapists. The second (qualitative) phase consisted of semi-structured interviews; the sample for this phase included 25 massage therapists, who were recruited from the sampling frame generated in the first phase.

### Survey – description of the study sites

Sixteen out of 305 hospitals identified in Canadian urban centers provided massage therapy services to patients by licensed massage therapists. The HBMTQ was emailed to all 16 sites. A total of 15 questionnaires were completed. The questionnaire was not completed at one site as permission from upper management for the clinic to be involved in the study was not received prior to the end date for data collection. Thirteen questionnaires (six managers and seven massage therapists) were completed by telephone; two respondents (one manager and one massage therapist) opted to complete it independently and submit it via fax and email.

The 15 study sites were located in four provinces across Canada (ten in Ontario, two in Alberta, two in Quebec, and one in Nova Scotia). Delivery of massage therapy services at the study sites spanned a period of 24 years. This included massage therapy services that have been provided between less than one and more than 20 years.

Six of the sites were categorized as teaching hospitals, five as large community hospitals, and four as small community hospitals. Nine of the sites were formally affiliated with a university. Eight of the sites provided focused areas of care, including HIV/AIDS, cancer, abdominal hernias, rehabilitation, pediatrics and women’s health. The remaining seven study sites provided various services and programs for a broad range of patient populations.

The number of patient beds at the sites ranged from 13 to 2424. One site had no beds as it functioned as an ambulatory care facility. The total number of staff across the sites ranged from 26 to 13,113. With the exception of one private hospital, all were public health care institutions.

In one third of the study hospitals, other complementary and alternative medicine (CAM) therapies were provided in the same setting as massage therapy. These included chiropractic, acupuncture, art therapy, music therapy, reflexology, reiki and therapeutic touch were provided by trained CAM practitioners, with chiropractic and acupuncture being most common.

### Interviews – perceptions of the massage therapists’ professional role

#### Participant recruitment

Forty-two massage therapists practiced across the 15 study sites surveyed, ranging from one to nine therapists per site. Given the low number of potential participants and our aim to generate a diverse sample, all were invited to participate in the interviews. Of those contacted, 25 massage therapists at 12 of the sites volunteered to participate in the study and all completed one telephone interview with the researcher (AKR).

#### Findings – the professional role of massage therapists

From the perspective of the massage therapists, their professional role as massage therapists encompassed a number of different components or aspects. The primary clinical role identified by the participants was that of a health care provider. However, a number of additional role components were also identified, which included: 1) Team member, 2) Program/clinic support, 3) Educator, 4) Promoter of the massage therapy profession, and 5) Researcher. A more detailed description of each is now provided.

#### Health care provider

As health care providers, massage therapists felt were expected to hold expert knowledge of specific techniques or modalities and provide massage interventions that were safe. Their responsibilities were to: assess patients, provide massage specific treatments, develop treatment plans, and report on their work and observations using formal and informal methods. Massage therapists were also expected to have a working knowledge of the patients’ condition(s) and related signs and symptoms, and more broadly the specific or unique needs and circumstances of that patient population. This is reflected in the experience of one massage therapist, who explained the various other factors that require attention and consideration:

*…working with HIV patients is really really unique…we have a lot of drug addiction issues and a lot of poverty issues… So for someone with poverty issues, they are struggling with how are they going to get here. So if they live downtown, they may not have money for a TTC token. So then we’ll provide tokens. But then sometimes that privilege will be abused. So then I have to evaluate, is this someone that can physically walk those two blocks? Or is it someone that needs a taxi chit. Or is it someone that has PMO and they are going to fall down and then I’m liable. Or is it someone that is just trying to get that taxi chit because they want it and always got it, but they’ll walk to their medical marijuana place… those are some of the challenges. (Site 1, Participant 1)*.

#### Team member

The nature of how massage therapists worked with others varied across the study sites. For almost all participants, working in a hospital setting involved some degree of interaction with other health care professionals (HCPs) in the context of patient care. However, only ten of the 25 participants (at half of the study sites) self-identified as team members. The indicators they used to determine their acceptance and/or recognition as members of a clinical or patient care team included documenting care in the same patient record as the rest of the team, access to all patient information, actively participating in and contributing to team meetings, receiving referrals from other clinicians, their clinical opinion being heard and taken into account by others, and consistent work hours.

Team participation involved various activities, yet two core components identified by almost all participants were: communication and care coordination.

#### Program/clinic support

Some study participants had an active role in developing and managing the massage therapy practice or the massage therapy component of a hospital program. This involved a range of responsibilities related to billing; organizing the massage therapists’ schedules and managing caseloads; liaising with supervisors or the hospital management to address massage therapy-specific issues; undertaking marketing and promotional activities, such as orientation sessions, distributing brochures, and, maintaining a website.

Massage therapists also undertook activities that were not directly related to the massage practice on their own accord. Some were engaged in activities to ensure the efficient functioning of the clinic or program within which massage therapy was integrated, such as developing and revising forms, developing program brochures, and planning activities to improve patient care and or team processes. One participant, in collaboration with a colleague (the recreational therapist), initiated and managed the development of a patient wellness program. This program aimed to provide a range of resources, including but not limited to CAM therapies, to support the physical, mental, social and financial well-being of long term patients upon discharge and re-entry into the community.

#### Educator

Many massage therapists reported they functioned as educators through formal and informal interactions with staff and patients. They organized and presented educational sessions for staff to build awareness about the massage therapy profession, the massage services available, and potential therapeutic benefits of massage treatments. In this capacity, participants reflected that they often clarified misconceptions or potentially incorrect labels about what massage therapy aims to do.

*“When I first started there, he (the doctor) said you must be happy to have your profession recognized. And I said what do you mean? And he said, “well, oh, it being an alternative profession”. And I said to him, “oh, well you may not be aware but we are part of the regulated health act in Ontario, the same as a nurse, physiotherapist, doctor”. So I think there was definitely a shift in perceptions over time about what we do”. (Site 2, Participant 2)*.

In the context of patient care, teaching patients exercises and activities of daily living was a common component of the massage treatments. Some participants also provided training to patients in application of specific massage techniques. For example, teaching self-massage to lymphedema patients as a self-care tool or teaching infant massage techniques to new parents.

Some participants were involved as educators in non-massage therapy related areas as well. For example, at one study site, the massage therapists were involved in providing education sessions for patients attending a specialized out-patient program on the health condition of concern, related risk factors, and recommendations for treatment.

#### Promoter of the massage therapy profession

Several massage therapists suggested that they were actively engaged in activities not only to market their services but also more broadly promote the massage therapy profession. Direct promotional activities were often tied to core business processes of the clinic or program to generate referrals and build or maintain active caseloads. A number of participants also recognize that their educational roles often overlapped into a promotional role where they used educational session for staff and patients as an opening to build awareness of and create support for the massage therapy profession within their respective settings.

Promoting the massage therapy profession also emerged through the development of a professional voice. Participants recognized that the ability to share their clinical opinions and be heard not only enabled them to contribute to patient care, but it was also perceived to help build the credibility of the massage therapy profession as a whole:

*“I speak up enough with my opinion …I think that how I conduct myself professionally makes it the product that it is. So if I was a therapist that attended rounds and didn’t say anything and didn’t add input and I didn’t even talk what it was that I was working on with a client, then I could be perceived as not a health care professional but a complementary person on staff. But it’s something that I feel um…like I always feel obligated to show that side because it’s important for the profession, a little bit, like for credibility”. (Site 1, Participant 1)*.

For one massage therapist, promoting the massage therapy profession meant using her experience of establishing a massage therapy program in a hospital to “open the doors” for its incorporation into other hospitals:

*“And the other role that I feel that I’m trying to fill is to widen the array of places where RMTs (registered massage therapists) could work. I would like to use the work that I am doing there to open up doors to as many hospitals as possible”. (Site 3, Participant 4).*

#### Researcher

Although several participants commented that they were involved in research processes to evaluate their practices, a true researcher role was only identified by two participants (P2 and P4). Both of the participants described activities related to initiating, developing, and undertaking research projects for the purpose of investigating specific outcomes of the massage therapy intervention they were providing. Neither of these participants had any formal research training and research was not a requirement or defined function of their position.

#### Mixed analysis – contextualizing themes across the study sites

Thematic findings, components of the massage therapists’ professional role, which emerged from the qualitative analysis presented up to this point, are based on the perceptions and experiences of all study participants. However, this presentation does not take into account or present how these themes may vary across the different study sites. Presenting the role components across the study sites within a descriptive matrix enables contextualization of the findings. This adds an additional comparative layer to the qualitative analysis, which enables a more in-depth understanding of massage therapists’ role. The mixed analysis is based on the 12 sites where both the questionnaire and interviews were completed.

Table 1 displays the distribution of the components of massage therapists’ professional role across the study sites. The main finding of the mixed analysis is a high degree of variability in how the role components manifested across the study sites. This suggests that how the professional role of massage therapists is defined and enacted by practitioners is context specific. Upon closer examination, however, several patterns are apparent.

**Table 1:** **Comparison of massage therapists’ role components across study sites**

	***Role components - Themes:***					
**Study site**	**Health care provider**	**Program/clinic support**	**Promoter**	**Educator**	**Team member**	**Researcher**
**S1**	√	√	√	√	√	
**S2**	√	√	√	√	√	√
**S3**	√	√	√	√		√
**S4**	√			√	√	
**S5**	√	√		√	√	
**S6**	√	√	√	√		
**S7**	√		√			
**S8**	√			√	√	
**S9**	√	√	√	√	√	
**S10**	√	√	√	√		
**S12**	√	√				
**S13**	√	√				

In considering each of the role components (columns), a clear trend is that the health care provider component was enacted at all of the sites. At more than half of the sites, massage therapists’ role appeared to include actively promoting the massage therapy profession (7/12), providing support (administrative and program development) in the functioning or running of the program where massage therapy was based (9/12), and carrying out various types of activities to educate others about massage therapy (9/12). Massage therapists were formally identified as members of patient care teams at half of the sites (6/12)*.* Researcher is the least common component of the massage therapists’ role, emerging at two sites. The high degree of variability in terms of the components which define the massage therapists’ professional role is also apparent when considering component combinations per site (Table 2). The full complement of role components occurred at only one site (site 2). The highest number of sites had a combination of four to five role components. At none of the sites was the health care provider the only component.

**Table 2:** **Configurations of massage therapists’ role components within study sites**

	***Role components – Themes:***					
**Study site**	**Health care provider**	**Program support**	**Promoter**	**Educator**	**Team member**	**Researcher**
**S2**	√	√	√	√	√	√
**S1**	√	√	√	√	√	
**S3**	√	√	√	√		√
**S9**	√	√	√	√	√	
**S5**	√	√		√	√	
**S6**	√	√	√	√		
**S10**	√	√	√	√		
**S4**	√			√	√	
**S8**	√			√	√	
**S7**	√		√			
**S12**	√	√				
**S13**	√	√				

## Discussion

This study aimed to provide a comprehensive descriptive account of hospital based massage therapy in Canadian urban hospitals. Of the Canadian urban hospitals that responded to the survey, sixteen (5%) organize and provide massage therapy services to patients by licensed massage therapists. The low number of hospitals was a reflection of the low prevalence of massage therapy in these settings. These hospitals (study sites) are located in four provinces: Alberta, Ontario, Quebec and Nova Scotia. The study sites encompassed a range of hospitals based on size, type, areas of care or patient population focus, and inclusion of other CAM therapies. Through the qualitative analysis, six components of the massage therapists’ professional role emerged *-* health care provider, educator, team member, program/clinic support, promoter of massage therapy profession, and researcher. It is important to note that the professional role of massage therapists’ is also formed and informed by the perceptions of others with whom the massage therapist interact in their role (e.g. patients, managers, colleagues). We recognized this in the conceptualization of the full study. Interviews with other health care professionals who worked with the massage therapists were also conducted to explore their perceptions of massage therapist’s professional role. For the purposes of this manuscript, the focus is on the perceptions of the massage therapists; findings from the interviews of other health care professionals will be presented separately in a subsequent publication by these authors.

Massage therapists’ perceptions of their professional role are comparable to the findings in previous studies in the health care professions literature for professions other than massage therapy. For instance, frequently cited components of a professional role are health care provider (or clinician) and educator [10,21-27]. Researcher was also a commonly cited role component in these studies and often part of the job description of the professionals. This is different from our findings, where massage therapists infrequently undertook research activities and when they did, it was not a requirement of their job or position. A number of other role components were also evident in the health professions literature. Important and distinct components of the nursing role which have been defined are liaison, patient care coordinator, team member [28], and leader [24]. Kinn and Aas [29] identified a number of components specific to the role of occupational therapists, such as: life coordinator, health agent, case manager, independent living specialist, and work trainer. Administrator was identified an important component of the physiotherapists’ role [30,31]. In a study on the perceptions of midwives on their roles, in addition to care provider, educator and evaluator of practice, Homer et al. [23] identified support provider and mentor as important components of the midwives’ role. As is evident, there is a range of role components that may be defined within a professional role. The fact that we identified a number of components for massage therapists is comparable to the other health care professions. In addition, there is overlap with some of these components (e.g. educator and team member); however, certainly not with all of the components that have been identified for the other HCPs.

Findings of the mixed analysis indicated that not all of the role components were encompassed within the massage therapists’ professional role at all of the sites. In fact, at only one site did the role of the massage therapists’ encompass all six components. With the exception of health care provider, there was variability in the configuration of role components which appeared to be site specific. However, at none of the study sites was the professional role perceived to consist of only the health care provider component. This suggests that although health care provider is a core component of the massage therapists role, their professional role appears to extend beyond massage treatment provision.

The variability of the remaining role components may results from a range of contextual factors, including expectations of massage therapists and other HCPs they work with, structures in the practice environment, and patient care processes. The variation or lack of consistency in the assumption of the various role components may also be indicative of the fact that, in general, the massage therapists’ professional role is not yet fully developed or clearly defined.

### Implications of the findings

The findings of this study have several important practice and education implications that warrant consideration and further discussion.

#### Practice implications

An important practice-related implication of this study is the direct engagement of the profession. Interactions with the study participants revealed that few had any connection or awareness of what was happening outside of their own setting in terms of hospital based massage therapy practice. Many were grappling with a range of similar issues in establishing their professional presence and some others which appeared to be context specific. Participation in the study resulted in identifying the fact that a number of massage therapists are engaged in hospital based practice and a point of departure for future collaborations in this area of massage therapy practice. In fact, three of the participants expressed interest in establishing a hospital-based practice network to support further developments in this area.

Working in a hospital setting requires some practical adaptations of massage practice from the standard approach used in the private health care setting [32,33]. However, hospitals also pose unique challenges which a massage therapist needs to be prepared to directly address. For instance, hospitals are settings dominated by power hierarchies and multiple boundaries between and within professions [9]. As such, massage therapists will likely find themselves in situations where they will need to negotiate their professional boundaries. This will require a balanced approach between advocating for their position and clearly delineating their abilities, skills, and areas of expertise, and adjusting their scope of practice in order to effectively work in a shared space (e.g. treatment space, similar knowledge) with other HCPs.

#### Education implications

For several participants, the broad question of “what is your role” was challenging to answer. They had difficulty articulating their role or what they did; as one participant reflected: *I don’t really think about it, I just do it* (Site 16, Participant 25). Others noted that expectations and responsibilities were not explicitly discussed or determined, particularly within multi-professional contexts. This lack of ability of some participants in this study to clearly articulate their role suggests that this important element may be currently missing in massage therapy training programs to adequately prepare massage therapists for practice. More specifically, this might mean that they have not been educated to reflect on their various roles, where their skills may overlap with those of other HCPs, and what their unique contribution to a multi-professional setting may be. This likely impedes their ability to establish themselves as team members in their own right; it might also be the reason that in some settings, their role is quite narrow.

As the findings of this study indicate, the incorporation of massage therapists’ role in a hospital setting is more than just a clinical issue. Massage therapists also function as, for example, educators and promoters of their profession. As such, competency development in these areas requires attention. More broadly, as massage therapy services are provided in a variety of health care settings, it is critical for massage therapists to graduate with the ability to adapt their practice to the different demands of these settings (as noted above). Competencies which reflect an understanding of the “culture, language, protocols and operations of institutional settings” [34], such as hospitals, are of particular importance for practitioners who are not exposed to and/or educated in conventional academic settings or exposed to conventional health care environments [34].

### Strengths and limitations of the study

This study addressed an important yet under-explored topic with practical relevance. Given the lack of research or information on hospital based massage therapy in Canada, the study required taking some risks. For instance, it was initially unknown how many hospitals provided massage therapy services and whether a sufficient sample size would be reached for a meaningful study. However, if advances are to be made into new areas of inquiry, such risks need to be taken. A key strength of this study was the application of a mixed methods design. The study design enabled a contextually based analysis of massage therapy roles, which enabled a more comprehensive exploration and one which will enhance the transferability of the findings to other hospital settings. Several strategies were used during data collection and analysis in both phases of the study to enhance the rigor of this study and trustworthiness of the findings. For example, the questionnaire was administered using a structured interview approach to ensure accurate data collection. In the qualitative phase, member checking and peer debriefing were two of the strategies employed to confirm findings.

The study had several limitations. The small sample size of hospitals (n = 16) precluded a statistically-based analysis to further assess the relationship between role themes and context. However, the contextualization of role themes in relation to study sites provides useful information as a starting point for consideration of how context may influence the creation and enactment of a professional role. The survey used to collect hospital descriptors was developed for the purposes of this study. During the course of data collection it became apparent that not all respondents had the information to respond to items on hospital descriptors (e.g. number of patient beds). Triangulation with interview data and hospital documents, when available, enabled confirmation of several questionnaire items.

### Future research

This study provides the first examination of massage therapy incorporation into Canadian hospitals. It may serve as a useful baseline or reference point for monitoring and evaluating ongoing developments in this area. In addition, the Hospital-Based Massage Therapy Questionnaire (HBMTQ) may be a useful tool to facilitate comparisons across hospitals and/or over time. The mixed analysis contextualizing the qualitative findings indicated that not all components of the massage therapists’ professional role identified were assumed at all of the study sites. This suggests that factors related to the practice setting may influence the establishment and enactment of massage therapists’ professional role. Future research may focus on further exploring how context factors impact professional role formation. Lastly, as already noted, the professional role of massage therapists is also informed through the perspectives of those who massage therapists interact with, for example the patients and other health care professionals. Further investigation of these perspectives is required to fully explore and map out the professional role of massage therapists.

## Conclusions

This is the first investigation of the incorporation of a popular CAM therapy, massage therapy, into Canadian urban hospitals. These findings make an important contribution in developing a comprehensive articulation of the massage therapists’ professional role. Although primarily exploratory, it provides a useful descriptive and contextually situated account of the massage therapy professional role in such a setting. A number of themes were identified, through which the multi-faceted nature of the massage therapists’ professional role emerged. The findings support the positive value of massage in patient care as perceived by massage therapists, and that there is a place for massage therapists in patient care in the hospital setting. This study enables massage therapists in hospital settings to better articulate their role and identify the different ways in which this service can be incorporated into hospital structures and processes.

## Additional files

Additional file 1:
**Urban Centers (Census Metropolitan Areas – CMAs) in Canadian provinces.** List of all urban centers in Canada where search for hospitals providing massage therapy services was conducted, based on data available through Statistics Canada. Additional file 2:
**Circulation of Research Notice - Massage Therapy Professional Associations and Regulatory Bodies.** List of organizations that agreed to circulate the research notice to their members. Additional file 3:
**Hospital Based Massage Therapy Questionnaire.** The questionnaire used in the study; results specific to Section 1 are presented for the purposes of this report. Additional file 4:
**Interview Guide – Role of Massage Therapists in hospital settings.** The interview guide used in the second (qualitative) phase of the study in the interviews with massage therapists.
