# Supplementary material for: The professional role of massage therapists in patient care in Canadian urban hospitals – a mixed methods study
Source: BMC Complement Altern Med. 2015 Feb 7;15:20. doi: 10.1186/s12906-015-0536-4 (PMC4355003; doi:10.1186/s12906-015-0536-4)
Supplement: Additional file 1: — Urban Centers (Census Metropolitan Areas – CMAs) in Canadian provinces. List of all urban centers in Canada where search for hospitals providing massage therapy services was conducted, based on data available through Statistics Canada. [file 12906_2015_536_MOESM1_ESM.pdf]

Additional File 1 - Urban Centers (Census Metropolitan Areas – CMAs) in Canadian Provinces

CITY (CMA)	PROVINCE		CITY (CMA)	PROVINCE
Abbotsford	BC		Peterborough	ON
Barrie	ON		Quebec City	QC
Brantford	ON		Regina	MN
Calgary	AB		Saguanay	QC
Edmonton	AB		St Johns	NB
Sudbury	ON		Saskatoon	Saskatchewan
Guelph	ON		Sherbrooke	QC
Halifax	NS		St. Catherine – Niagara Falls	ON
Hamilton	ON		St John	NFLD
Kelowna	BC		Thunder Bay	ON
Kingston	ON		Toronto	ON
Kitchener	ON		Trois Riviere	QC
London	ON		Vancouver	BC
Moncton	NB		Victoria	BC
Montreal	QC		Windsor	ON
Oshawa	ON		Winnipeg	MN
Ottawa	ON			

Based on data from Statistics Canada:

Statistics Canada: **Census metropolitan area (CMA) and census agglomeration (CA).** Statistics Canada Online Catalogue no. 98-301-XWE. Ottawa. 2012. www12.statcan.gc.ca/census-recensement/2011/ref/dict/geo009-eng.cfm
