# Supplementary material for: The professional role of massage therapists in patient care in Canadian urban hospitals – a mixed methods study
Source: BMC Complement Altern Med. 2015 Feb 7;15:20. doi: 10.1186/s12906-015-0536-4 (PMC4355003; doi:10.1186/s12906-015-0536-4)
Supplement: Additional file 2: — Circulation of Research Notice - Massage Therapy Professional Associations and Regulatory Bodies. List of organizations that agreed to circulate the research notice to their members. [file 12906_2015_536_MOESM2_ESM.pdf]

Additional File 2 - Circulation of Research Notice - Massage Therapy Professional Associations and Regulatory Bodies

- Massage Therapy Association of Saskatchewan
- Massage Therapists' Association of Alberta
- Massage Therapists Association of British Columbia
- Federation Quebecoise Des Massotherapeutes Agrees
- Registered Massage Therapists Association of Ontario
- College of Massage Therapists of Ontario
