# Supplementary material for: The professional role of massage therapists in patient care in Canadian urban hospitals – a mixed methods study
Source: BMC Complement Altern Med. 2015 Feb 7;15:20. doi: 10.1186/s12906-015-0536-4 (PMC4355003; doi:10.1186/s12906-015-0536-4)
Supplement: Additional file 3: — Hospital Based Massage Therapy Questionnaire. The questionnaire used in the study; results specific to Section 1 are presented for the purposes of this report. [file 12906_2015_536_MOESM3_ESM.pdf]

Additional File 3: Hospital Based Massage Therapy Questionnaire (English version)

Completed by: ____

Position:____ Date: ________________________

SECTION 1: DELIVERY OF MASSAGE THERAPY SERVICES

Question 1-1: What areas of the hospital are massage therapy services incorporated in? Check all that apply:

- ☐ Department or unit, name:____
- ☐ Clinic, name: ____
- ☐ Institute, name:____
- ☐ Program, name:____
- ☐ Wellness or CAM center, name: ____
- ☐ Stand alone massage therapy clinic: ____
- ☐ Other, please specify: ____

Question 1-2: In addition to massage therapists, what health care professionals provide patient care in this/these areas? Check all that apply:

	**Area 1**	**Area 2**	**Area 3**	**Area 4**
Doctor				
Nurse				
Physiotherapist				
Occupational Therapist				
Social worker				
Psychologist				
Nutritionist/dietician				
Speech Language Pathologist				
Pharmacist				
Spiritual care provider				
Respiratory therapist				
Recreational therapist				
Midwife				
Other, please specify:				

Question 1-3: **How long has massage therapy been provided at your hospital?**

- ☐ _____________ (years)
- ☐ _____________ (other)
- ☐ Do not know
- ☐ Other:

Question 1-4*:* **Who has access to the massage therapy services in your hospital? Check all that apply:**

- ☐ In-patients
- ☐ Out-patients – individuals attending a specific hospital program
- ☐ Community clients
- ☐ Hospital employees
- ☐ Other, please specify: ___________________________________
- ☐ Do not know

Question 1-4b – Of the massage therapy client groups indicated in question 1.4, indicate the percentage each makes up out of all massage therapy clients:

- ☐ In-patients - % of all MT clients: ______________
- ☐ Out-patients - % of all MT clients: ____________
- ☐ Community clients - % of all MT clients:_____________________
- ☐ Hospital employees % of all MT clients:________________
- ☐ Other, please specify - % of all MT clients: ___________________________________
- ☐ Do not know

Question: 1-5: Approximately what percentage of all patients (in-patients and out-patients) receives massage therapy?

- ☐ _______________________

Question 1-6: What are the reason(s) for providing massage therapy at your hospital? Check all that apply:

- ☐ Holistic philosophy of care of the institution
- ☐ Revenue generation
- ☐ Patient satisfaction
- ☐ Patient demand
- ☐ Evidence on the effectiveness of MT
- ☐ Employee request
- ☐ Physician request
- ☐ Other health care providers' request
- ☐ Other, please specify: ________________________________

Question: 1-7: How are patients informed about massage therapy at your hospital? Check all that apply:

- ☐ At admission – admission materials regarding hospital programs and services
- ☐ Advertising – internal (brochures, screen ads, hospital newsletter)
- ☐ Health care providers
- ☐ Other, please specify: ___________________________

Question 1-8: Where are massage therapy services/treatments provided in your hospital? Check all that apply:

- ☐ Patient room
- ☐ Therapy room
- ☐ Designated massage therapy space (room, clinic)
- ☐ Wellness/CAM center
- ☐ Other, please specify:____

Question 1-9: When are massage therapy services provided at your hospital? Check all that apply:

- ☐ Monday to Friday- day (9-5pm)
- ☐ Monday to Friday – evenings (after 5pm)
- ☐ weekend
- ☐ Statutory holidays
- ☐ Other: please specify:____

Question 1-10: How many massage therapists are currently working at your hospital?

- ☐ _____________

Question 1- 11: What is the employment status of the massage therapist(s) at your hospital? Check all that apply:

- ☐ hospital employee
    - o full time
    - o part time
    - o casual
- ☐ independent contractor
- ☐ other: ______________________

Question 1-12: How many hours of treatment does each massage therapist provide per week (on average) at your hospital?

- ☐ less than 5
- ☐ 6-10
- ☐ 11-15
- ☐ 16-20
- ☐ 21-25
- ☐ more than 25
- ☐ exact number:________________
- ☐ don't know

Question 1-13: What is the fee schedule for massage therapy services at your hospital? Indicate all that apply:

- ☐ 15 mins _$__________
- ☐ 30 mins - _$_________
- ☐ 45 mins - $__________
- ☐ 60 mins - _$_________
- ☐ 90 mins -_$_______
- ☐ No charge
- ☐ other:____

Question 1-14: How are massage therapy services funded (financed) at your hospital? Check all that apply:

- ☐ By the patient - out of pocket
- ☐ 3rd party (private insurance) – extended health care (e.g. employer; blue cross)
- ☐ 3rd party (Private insurance) -car insurance (motor vehicle accident insurance),
- ☐ 3rd party (private insurance) – workers' compensation
- ☐ Global hospital budget
- ☐ Charity/charitable fund – external
- ☐ Hospital foundation – internal
- ☐ Specific project or grant
- ☐ No charge - provided on a volunteer basis
- ☐ Other:____

Question 1-15: How are massage therapists reimbursed for the services they provide at your hospital? Check all that apply:

- ☐ By the hospital
- ☐ Directly by the patient
- ☐ By patient and the hospital
- ☐ Directly by 3rd party insurance
- ☐ Invoice the hospital
- ☐ Other, please specify: ____

Question 1-16: How and what amount are massage therapists paid at your hospital?

- ☐ per hour: _$__________
- ☐ per treatment: _$__________
- ☐ per treatment: split of MT fee: ____________________
- ☐ other:______________

Question 1-17: What qualifications do massage therapists need to have in order to provide massage therapy services at your hospital? Check all that apply:

- ☐ License to practice
- ☐ Practice and liability insurance
- ☐ 2200-hour training completed
- ☐ Other, please specify: _______________________________________

Question 1-18: Who is responsible for verifying the credentials of massage therapists at your hospital? Check all that apply:

- ☐ Human resource personnel
- ☐ Program/unit manager or director
- ☐ Professional practice leader (PPL)
- ☐ Other, please specify: ______________________________

Question 1-19: In addition to massage therapists, who else provides massage therapy services at your hospital? Check all that apply:

- ☐ Massage therapy students
- ☐ Nurses
- ☐ Physiotherapists
- ☐ Other, please specify:___________________
- ☐ None (only licensed massage therapists)

Question 1-20: Who can refer to massage therapy at your hospital? Check all that apply:

- ☐ Patient - self-referral
- ☐ Internal referral - physician
- ☐ Internal referral - nurse
- ☐ Internal referral – allied health professionals
- ☐ External referral - physician
- ☐ External referral - nurse
- ☐ External referral – allied health professional
- ☐ Other, please specify:____

Question 1-21: Is a doctor's order required for a patient to receive massage therapy at your hospital?

- ☐ Yes
- ☐ No

Question: 1-22: Do massage therapists have access to patient charts at your hospital?

- ☐ Yes
- ☐ No

Question: 1-23: On which chart(s) do massage therapists document regarding the patient care they provide at your hospital? Check all that apply:

- ☐ Multi-disciplinary/team charts
- ☐ Independent massage therapy charts
- ☐ Other: ______________________

Question 1-24: Do massage therapists contribute to patient reports at your hospital?

- ☐ Yes
- ☐ No

Question 1-25: Do massage therapists participate in meetings related to patient care at your hospital?

- ☐ Yes
- ☐ No

Question 1-26: Are massage therapists members of patient care teams at your hospital? Check one:

- ☐ Yes – go to question 1-27
- ☐ No - go to question 1-28

Additional comments:

Question 1-27: What healthcare providers are on the patient care teams where massage therapists are included? Check all that apply:

- ☐ Doctor
- ☐ Nurse
- ☐ Physiotherapist
- ☐ Occupational Therapist
- ☐ Social worker
- ☐ Psychologist
- ☐ Nutritionist/dietician
- ☐ Speech Language Pathologist
- ☐ Pharmacist
- ☐ Spiritual care provider
- ☐ Respiratory therapist
- ☐ Recreational therapist
- ☐ Midwife
- ☐ Other, please specify:_______________________________

Question 1-28: Do you have any other thoughts or comments about massage therapy at your hospital? Please write them in the space provided below:

SECTION 2 – HOSPITAL DESCRIPTORS

Question 2-1: What type of hospital do you operate? Check all that apply:

- ☐ Teaching hospital
- ☐ Small community hospital
- ☐ Medium community hospital
- ☐ Large community hospital
- ☐ Other, please specify:_____________________________________
- ☐ Do not know

Question 2-2: Does your hospital have an area (or areas) of specialization? Check all that apply:

- ☐ Chronic care
- ☐ Psychiatric/mental health
- ☐ Alcohol and drug addiction
- ☐ Rehabilitation
- ☐ Women's health
- ☐ Children's/pediatrics
- ☐ Military
- ☐ Convalescent
- ☐ Other, please specify:_____________________________________
- ☐ Do not know

Question 2-3: Is the hospital affiliated with a university?

- ☐ No
- ☐ Yes, please provide university name: _________________________________
- ☐ Do not know

Question 2-4: What is the business model of your hospital? Check one:

- ☐ Not-for-profit
- ☐ For profit
- ☐ Other, please specify: ____
- ☐ Do not know

Question 2-5: What is the annual budget of your hospital? Check one:

- ☐ Under $10 million
- ☐ Over $10 million to $100 million
- ☐ Over $100 million to $500 million
- ☐ Over $500 million to 1 billion
- ☐ Over $1 billion
- ☐ Other: __________________
- ☐ Do not know

Question 2-6: What is the total number of designated patient beds in your hospital? Check one:

- ☐ Less than 100
- ☐ 101-400
- ☐ 401-800
- ☐ Over 800
- ☐ Other:________________
- ☐ Do not know

Question 2-7: What is the number of clinical staff at your hospital?

- ☐ ______________
- ☐ Do not know

Thank you for your time in completing the

Hospital-Based Massage Therapy Questionnaire

**For the French versions, please contact the corresponding author*
