# Supplementary material for: The professional role of massage therapists in patient care in Canadian urban hospitals – a mixed methods study
Source: BMC Complement Altern Med. 2015 Feb 7;15:20. doi: 10.1186/s12906-015-0536-4 (PMC4355003; doi:10.1186/s12906-015-0536-4)
Supplement: Additional file 4: — Interview Guide – Role of Massage Therapists in hospital settings. The interview guide used in the second (qualitative) phase of the study in the interviews with massage therapists. [file 12906_2015_536_MOESM4_ESM.pdf]

Additional File 4: Interview Guide – Role of Massage Therapists in hospital settings

1. Can you describe for me the where you work in the hospital (as a massage therapist)?
2. How long have you worked there?
3. Have you ever worked in a hospital setting before?
4. Do you work with any other providers/health care professionals?
    - Which health care professionals?
5. Are you part of a multi or inter-professional clinical team within the hospital?
    - Who makes up the team (who are the other team members)?
6. Based on your experience, what is your role as a massage therapist in patient care?
    - Probes: skills, activities/tasks, responsibilities, expectations, knowledge
    - Is there anything that you could do in relation to patient care that you are currently not doing or unable to do?
7. What do you contribute in your role as a massage therapist to patient care?
8. How is your role as a massage therapist determined? By whom?
9. Does your role overlap with any of the health care professionals?
    - In what ways does it overlap?
    - How is this dealt with?
10. Have you encountered any challenges in your role as a massage therapist?
    - Probes: difficult moments, conflicts, uncomfortable situations, unclear about what to do
